# Multiple evanescent white dot syndrome and panuveitis: a case report

**DOI:** 10.1186/s12348-020-00221-3

**Published:** 2020-10-29

**Authors:** Kazuomi Mizuuchi, Wataru Saito, Kenichi Namba, Susumu Ishida

**Affiliations:** 1 Department of Ophthalmology, Faculty of Medicine and Graduate School of Medicine, Hokkaido University, Nishi 7, Kita 15, Kita-ku, Sapporo, 060-8638 Japan; 2 Kaimeido Eye and Dental Clinic, Sapporo, Japan

**Keywords:** Choroiditis, Iridocyclitis, Multiple evanescent white dot syndrome, Panuveitis, Vitritis

## Abstract

**Aim:**

To report a patient with multiple evanescent white dot syndrome (MEWDS) complicated by iridocyclitis and vitritis.

**Case description:**

A 70-year-old woman developed multiple subretinal white dots, iritis, and diffuse vitreous opacity. Angiographic and macular morphological features were consistent with those of MEWDS. Inflammatory findings including the white dots improved following only topical dexamethasone within 1 month after the initial visit. Best-corrected visual acuity recovered to 1.0 with restored photoreceptor structure.

**Conclusion:**

The presence of iridocyclitis and vitritis, atypical to MEWDS, indicates the concurrent development of panuveitis associated with MEWDS. These results suggest that MEWDS is a clinical entity of uveitis.

## Background

Multiple evanescent white dot syndrome (MEWDS) is a unilateral inflammatory disease characterized by multiple subretinal white dots extending from the posterior pole to the mid-periphery, which spontaneously resolve with no scaring. In eyes with MEWDS, anterior chamber and/or anterior vitreous cell infiltration can occur; however, the grade of inflammation is usually mild [1]. We herein report a rare case of MEWDS complicated by iridocyclitis with diffuse vitreous opacity.

## Case report

A 70-year-old woman presented with a month-long history of blurred vision and photopsia of her left eye. She had medical history of well-controlled systemic hypertension and hyperlipidemia and had no particular family history.

**Fig. 1:**
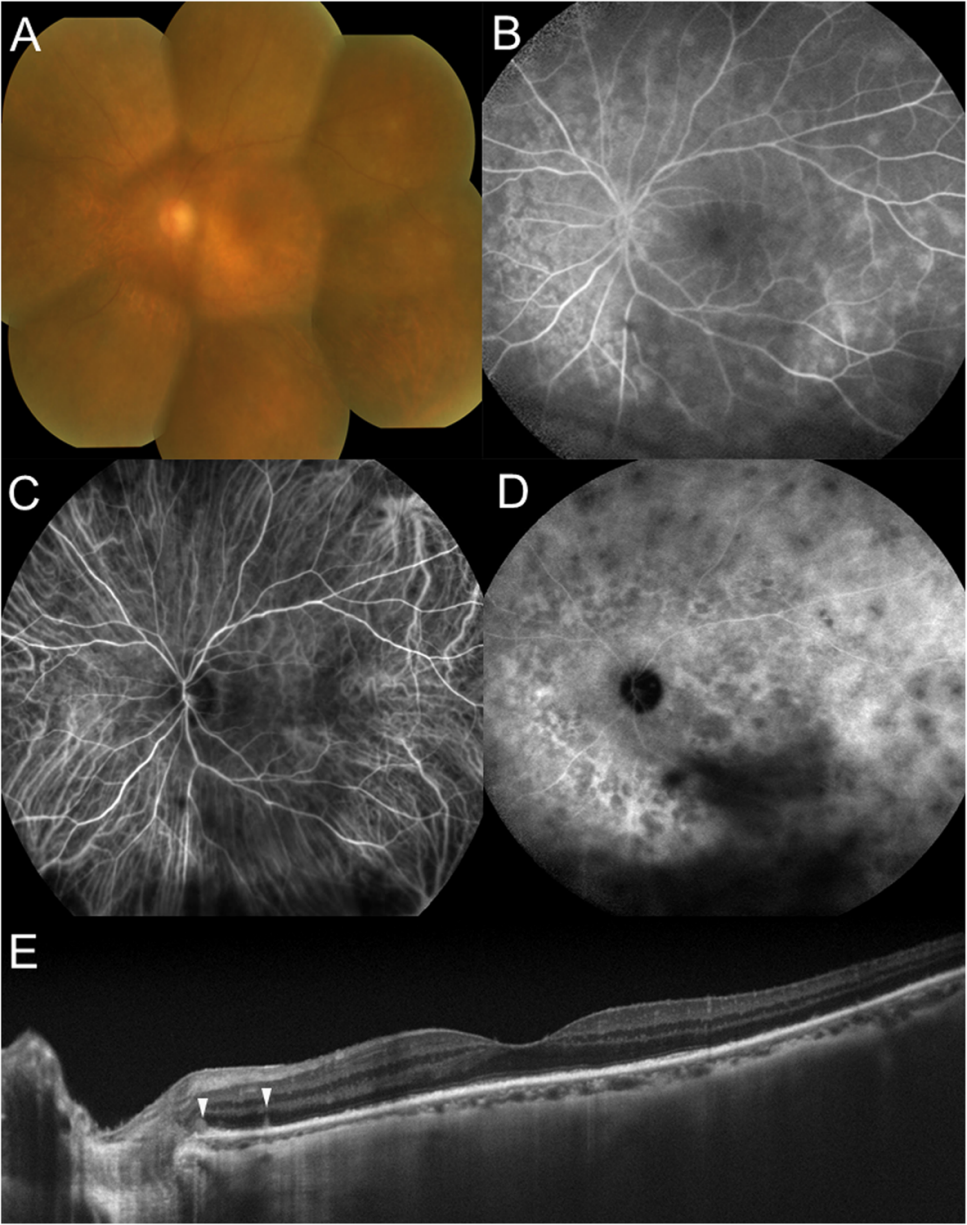
Findings of the left eye at the initial visit in a 70-year-old woman with multiple evanescent white dot syndrome with panuveitis. **a** Fundus was seen hazy due to diffuse vitreous opacity. Multiple subretinal white dots at the mid-periphery could be barely observed. **b** Corresponding to the white dots, fluorescein angiography (FA) 2 min. after the dye injection showed hyperfluorescence as well as at the peripapillary area. **c, d** Indocyanine green angiography (ICGA) showed no abnormal findings 1 min. after the dye injection (**c**) and numerous hypofluorescent dots extending from the peripapillary area and posterior pole to the mid-periphery 16.5 min. after the dye injection (**d**). Inferior black shadow resulted from the vitreous opacity. **e** A horizontal image through the fovea on enhanced depth imaging optical coherence tomography (EDI-OCT) showed diffuse loss of the ellipsoid zone at the macula and hyper-reflective foci at the level of the photoreceptor (arrowheads)

**Fig. 2:**
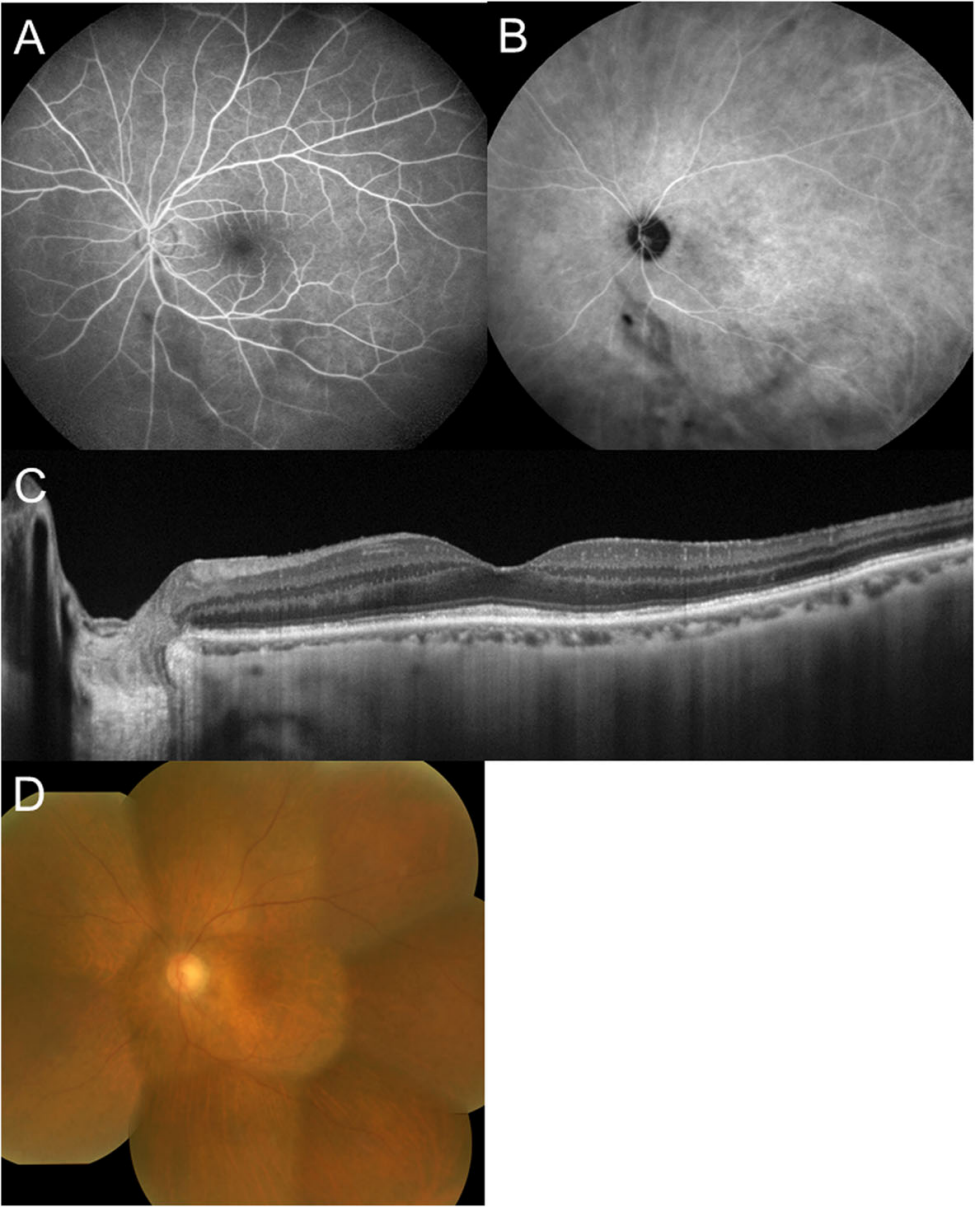
Findings of the left eye 2 weeks (**a, b**) and 3 weeks (**c, d**) after the first visit in the present case. **a** On FA 2 min. after the dye injection, hyperfluorecence observed at the initial visit disappeared. **b** On ICGA 16.5 min. after the dye injection, numerous hypofluorescent dots regressed. **c** EDI-OCT revealed the recovery of macular ellipsoid zone and absorption of hyper-reflective foci. **d** Color fundus photography showed resolution of the white dots with no chorioretinal scaring

Her best-corrected visual acuity (BCVA) was 1.0 OD and 0.3 OS with mild hyperopia. The right eye was normal except for incipient cataract. Slit-lamp examination revealed 1+ flare and 1+ cells in the anterior chamber together with the infiltration of anterior vitreous cells OS. Her left fundus was hazy due to 2+ diffuse vitreous opacity; and multiple subretinal white dots could thus be barely observed at the mid-periphery (Fig. 1a). Corresponding to the white dots, fluorescein angiography (FA) showed hyperfluorescence as well as at the peripapillary area in the late phase (Fig. 1b) and indocyanine green angiography (ICGA) revealed no abnormal findings in the initial phase (Fig. 1c) but numerous hypofluorescent dots were visible not only at the white dots but also diffusely around the posterior pole including the optic disc vicinity in the late phase (Fig. 1d). Optical coherence tomography (OCT) showed diffuse loss of the ellipsoid zone (EZ) at the macula OS (Fig. 1e). Goldmann perimetry revealed a ring scotoma of 50 × 70 degrees OS. Single-flash electroretinography showed a reduced amplitude of the a-wave OS. Screening tests were negative for the detection of syphilis, tuberculosis, and sarcoidosis. The patient was followed up with only topical 0.1% betamethasone under a diagnosis of presumed MEWDS OS, although the presence of vitritis was atypical for MEWDS. Five days later, the number of the white dots, anterior chamber inflammation, and diffuse vitreous opacity were reduced. Seventeen days after the first visit, her BCVA improved to 1.0. Hyperfluorescence on FA and hypofluorescent dots on ICGA initially observed almost disappeared (Fig. 2a, b). Three weeks after the first visit, macular EZ loss improved (Fig. 2c), followed by the regression of diffuse vitreous opacity and subretinal white dots OS (Fig. 2d). Electroretinography and perimetry normalized after 3 months. Brain magnetic resonance imaging and systemic computed tomography revealed no abnormal findings. There were no recurrences of intraocular inflammation until 63 months after the initial visit. Her BCVA maintained at 1.0 OD and 0.9 OS.

## Discussion

We encountered a case of atypical MEWDS complicated by iridocyclitis with diffuse vitreous opacity. The presence of blurred vision with photopsia, unilateral multiple subretinal white dots, features of FA, ICGA, and OCT at the acute stage was consistent with the features of MEWDS. Moreover, resolution of the white dots with no scaring, recovery of OCT, angiographic, and electroretinographic findings, and favorable visual prognosis with no recurrence of intraocular inflammation for a long-term follow-up period supported a diagnosis of MEWDS. On the other hand, the presence of intraocular inflammation with vitritis, the onset at the advanced age, and the development in a hyperopic eye were atypical for MEWDS.

Diseases masquerading as MEWDS are considered as the differential diagnosis of this case [2]. In the present case, ICGA showed initial normal and late numerous dot-shaped hypofluorescent appearances extending from the peripapillary area and posterior pole to the mid-periphery containing funduscopically visible white dots, which rapidly resolved with no scarring. The features were characteristic of MEWDS [3] and were inconsistent with those of white dot syndrome other than MEWDS including multifocal choroiditis and birdshot chorioretinopathy (hypofluorescence from the initial phase and the development of scar lesions with time corresponding to exudates). Syphilitic chorioretinitis was denied from negative infectious serology. In patients with intraocular lymphoma, the recurrence of ocular involvement is common and the survival rate is poor [4], even if ocular lesions initially respond with systemic corticosteroid treatment. In our case, favorable visual and systemic prognosis with no recurrence of intraocular inflammation for a long-term follow-up period is incompatible with the clinical course of intraocular lymphoma, although vitreous biopsy was not performed. Similarly, cancer-associated retinopathy is unlikely to be considered, although anti-retinal antibodies were not examined.

The localization and origin of subretinal white dots in MEWDS is still controversial. Studies using fundus autofluorescence have revealed hyper-autofluorescence corresponding to the white dots at the acute stage of MEWDS, suggesting impairment of the retinal pigment epithelium [5]. Previous studies showed hypo-reflective lesions at the level of the photoreceptors but no abnormal findings at the choriocapillaris on en face OCT with flow preservation at the choriocapillaris on OCT angiography corresponding to the white dots [6, 7]. These observations may indicate that the photoreceptor is primarily affected in MEWDS. In eyes with MEWDS, multiple hypofluorescent dots observed on ICGA suggest the involvement of hypoperfusion in the choriocapillaris [3]. In white dot syndromes other than MEWDS, such as serpiginous choroiditis, multifocal choroiditis (MFC) and panuveitis, and acute posterior multifocal placoid pigment epitheliopathy (APMPPE), OCT angiography demonstrated the reduction of choriocapillaris flow [7], suggesting the choroid as a susceptible lesion targeted in these diseases. On the other hand, contradictory results on OCT angiography (i.e., reduced versus preserved flow void at the choriocapillaris) have been reported in eyes with MEWDS [6–8]. Moreover, most recent studies using OCT angiography demonstrate that flow void at the choriocapillaris corresponding to the white dots of MEWDS and significant reduction in the vascular density of the choriocapillaris at the macula [9, 10], suggesting the existence of circulatory disturbance at the choriocapillaris in MEWDS. Taken together, lesions in MEWDS may at least lie not only in the photoreceptors but also in the RPE and choriocapillaris. Further studies with large number of cases, using OCT angiography, are needed to verify the involvement of circulatory disorder of the choriocapillaris in MEWDS.

In parallel with these angiographic analyses, we have recently demonstrated two distinct but relevant manifestations of the choroid at the acute stage of MEWDS: “choroidal thickening with reduced blood flow”, both of which correlated with visual function [11, 12], suggesting a close link between choroidal circulatory disturbance and visual impairment in MEWDS. Moreover, we identified the inner layer of the choroid as a changeable area responsible for whole choroidal thickening seen at the acute stage of this disease [13], indicating a possibility of the choriocapillaris as the primary focus of MEWDS. The combination of choroidal thickening with impaired circulation would strongly suggest the involvement of choroiditis in the pathogenesis of MEWDS, according to our previous analyses of uveitic diseases such as Vogt-Koyanagi-Harada disease [14] and white dot syndromes including serpiginous choroiditis, punctate inner choroidopathy (PIC), and APMPPE [15–17].

Consistently, a recent case series of MEWDS revealed a negative correlation between reduced intraocular pressure (IOP) and elevated aqueous flare at the acute stage, suggesting the potential role of subclinical cyclitis in compromising aqueous humor production and thus lowering IOP in eyes with MEWDS [18]. The present case developed severer iridocyclitis and vitritis than typical MEWDS does, possibly because inflammation uncommonly extended not only to the choroid but to the whole uvea. MEWDS would reasonably be to our atypical case what PIC is to ‘MFC and panuveitis’, a spectrum disorder with identical etiology but greater severity [19]. Therefore, our atypical case may be called ‘MEWDS and panuveitis’, suggesting that MEWDS is a clinical entity of uveitis.

## Data Availability

All data generated or analysed during this study are included in this published article.

## Abbreviations

MEWDS: Multiple evanescent white dot syndrome
BCVA: Best-corrected visual acuity
FA: Fluorescein angiography
ICGA: Indocyanine green angiography
OCT: Optical coherence tomography
MFC: Multifocal choroiditis
APMPPE: Acute posterior multifocal placoid pigment epitheliopathy
